# Globalization, platform work, and wellbeing—a comparative study of Uber drivers in three cities: London, Helsinki, and St Petersburg

**DOI:** 10.1186/s12992-024-01021-3

**Published:** 2024-03-01

**Authors:** Meri Koivusalo, Arseniy Svynarenko, Benta Mbare, Mikko Perkiö

**Affiliations:** Tampere University, Tampere, FI-33014 Finland

**Keywords:** Globalization, Platform work, Wellbeing, Psychosocial stress, Regulation, Uber

## Abstract

**Background:**

Globalization of platform work has become a challenge for wider social and employment relations and wellbeing of workers, yet on-location work remains governed also by local regulatory context. Understanding common challenges across countries and potential for regulatory measures is essential to enhance health and wellbeing of those who work in platform economy. Our comparative study on platform work analyzed concerns of Uber drivers in three cities with a different regulatory and policy context.

**Methods:**

Drawing from current understanding on employment and precarity as social determinants of health we gathered comparative documentary and contextual data on regulatory environment complemented with key informant views of regulators, trade unions, and platform corporations (*N* = 26) to provide insight on the wider regulatory and policy environment. We used thematic semi-structured interviews to examine concerns of Uber drivers in Helsinki, St Petersburg, and London (*N* = 60). We then analysed the driver interviews to identify common and divergent concerns across countries.

**Results:**

Our results indicate that worsening of working conditions is not inevitable and for drivers the terms of employment is a social determinant of health. Drivers compensated declining pay with longer working hours. Algorithmic surveillance as such was of less concern to drivers than power differences in relation to terms of work.

**Conclusions:**

Our results show scope for regulation of platform work especially for on-location work concerning pay, working hours, social security obligations, and practices of dismissal.

**Supplementary Information:**

The online version contains supplementary material available at 10.1186/s12992-024-01021-3.

## Background

Precarious employment and its role as a social determinant of health has been under scrutiny already before the wider emergence of platform work [1]. We use de Groen et al. (2015) definition [2]: “Platform work is paid work that is organized through an online platform to carry out specific tasks, solve problems, or to provide services in exchange for payment with the following main characteristics: “Three parties are involved: the online platform, the client and the worker; the work is outsourced or contracted out; jobs are broken into tasks; services are provided on demand.” This is close to the definition used by European Commission [3]: “Platform work is a form of employment in which organisations or individuals use an online platform to access other organisations or individuals to solve specific problems, or to provide specific services in exchange for payment.​”.

Platform work includes both online and on-location work. Uber provides on-location work in the form of ride-hailing and delivery services. In this article we focus on ride-hailing in the wider European comparative context.

The expansion of precarious employment and platform work has drawn research interest to conditions of labor and employment in global and local contexts [4–5]. Studies on platform work associate it with vulnerability to work insecurity, unpredictability of work, low wages, and income insecurity [6–9]. These are all associated with poor mental health, including stress, depression, and anxiety [6–7, 10].

Uber and other platform companies have become crucial players in globalization and global capitalism [11–12]. Uber provides a mobile app, which is used to order and pay for different types of rides- from luxury cars to budget cars, and currently also provides delivery services using map-based location. In the initial model, drivers use their own cars and operate as self-employed. Uber operates in more than 10 000 cities with 5.4 million drivers and delivery workers [13]. It has global headquarters in San Fransisco and for Europe, Middle East, and Africa in Amsterdam. Uber fits well to a description of a globalized corporation having globalized its operational business model across countries.

Due to Uber’s major corporate “disruptor” role, societal impacts of platform economy have become addressed as “uberisation” [11, 14]. Uber has also drawn widespread attention as result of its influence on policymakers. Investigative journalism of leaked “uberfiles” have showed how Uber engaged widely with European policymakers, including with European Union, Finnish, and Russian decision-makers [15–17]. While global platform companies would benefit from international regulatory framework, regulation of industries has so far taken place more at local level and through court cases. Thus, our study was set in the globalization of labour market conditions and in the intersection between a global corporation and diverse national regulatory environments. The comparative study in three different contexts enabled to focus on commonalities and differences across for Uber drivers views across different regulatory contexts.

Platformisation influences job quality and many “gig economy jobs” can be seen in the continuum of precarisation of work. While the ILO has emphasized focus on decent work, this concept has been applied less in academic research and literature [18]. However, prior research on platform work has covered in practice dimensions of precarity and how digitalisation and algorithmic management relate to the lived experience and wellbeing of platform workers in studies on Uber drivers in the United States [8, 19–22], Australia [23–24], EU and UK [24–29]. We have comparative work between Uber and other platform providers [19, 21, 29], but we have less comparative work across countries for driver interviews [21, 22]. We did not include in our driver interviews detailed focus on social protection but covered this more as part of focus on context. In terms social protection many concerns in the gig economy resonate with situations of other non-standard workers [30, 31]. Myhill et al. (2021) used a Fairwork Convention for assessment of job quality in Scotland with rather similar thematic emphasis [29]. Our focus on job quality follows wider frameworks and domains on decent work and quality of work and employment, which mostly draw from survey or indicator data [32–34]. However, as our interest was on the gig-work we included focus on algorithmic management and psychosocial stress as part of the interviews and analysis.

Platformisation can be seen as a social determinant of health [35]. However, impacts of “uberisation” have not been recorded for overall accident rates or fatalities [36–37]. Health implications from platformisation and algorithmic management are thus not necessarily mediated by accident rates or immediate physical working conditions for the drivers. On the other hand, there is increasing interest in the psychosocial stress and changing conditions of work [8, 38], including “technostress” [39]. Our approach and thematic analysis of the interviews was thus also informed by the more general demand-control (DC) [40] and effort-reward (ER) imbalance [41] models on psychosocial stress.

Platformisation and algorithmic management have relevance to changes in working and employment conditions [9, 15–16]. Flexibility of when to work has been appreciated by drivers [7]. However, economic dependency from platform work (i.e. platform work as main not supplementary income) has been shown to be of importance across different types of platform workers [21]. Implications on psychosocial health and wellbeing are likely to reflect also wider social context. Welfare regimes matter for non-standard work [42–43]. For example, in contrast to United States, shifting costs for access to health services from “employer” to “worker” has less importance in Finland and United Kingdom with national health services covering main costs of services. In this respect our work complements insights of prior comparative work on regulatory context between US and Europe and within Nordic countries [44–45]. Understanding social, political, and regulatory context of platform work and their implication to wellbeing in the wider societal and regulatory context is thus crucial [42–43, 46–47].

Our wider theoretical framework for analysis thus draws from the understanding that drivers’ expectations and views are influenced by their personal experiences and background, global corporate practices, and local regulatory context. Through the focus of one global platform corporation (i.e. Uber), it would be possible to tease out from the driver interviews those aspects, which were common across countries as well as elements, which were more related to the particular context in which and by whom the platform work took place allowing to focus in particular on how platform work relates to globalization and health.

## Materials and methods

Our project RRR-Uber had its focus on three cities– Helsinki (Finland), London (the UK), and St Petersburg (Russia)– where we interviewed twenty (20) drivers in each city who used a ride-haling app as the main instrument for finding clients. Our driver interviews were initiated in Fall 2019 in St Peterburg and finalized in June 2021 in London due to some delay as result of the Covid-19 pandemic. Drivers interviewed predominantly identified as men with one female driver (1/20) in Helsinki and London (Table 1.)

**Table 1:** Sociodemographic description of drivers interviewed in each city

Location	Helsinki, capital of Finland, 0.7 million residents	Saint Petersburg, second-largest city in Russia, 5.4 million residents,	London, capital of the UK, 9.9 million residents.
Number of interviews	20	20	20
Field work period	01/ 2020-06/2020	12/2019- 02/2020	04/ 2021-06/2021
Average age of informants (out of/20)	43 (19/20)	37(17/20)	45 (19/20)
Average duration of interview, minutes	37	30	52
Uber/Yandex driving as main job	16/20	12/17	17/19
Ride-hailing apps used for finding informants	Uber	Yandex.Taxi	Uber
Other	on-location	on-location	video/voice call
University level education	12/20 University degree	8/19 University degree	4(2)/18 University degree (incomplete)
Mode of work (entrepreneur)	entrepreneurs	in-between employee (from control/autonomy perspective) and self-employment (bare full responsibility for welfare) status	entrepreneurs

Drivers were recruited by purposeful sampling and on a voluntary basis. To increase reliability, we paid close attention to covering different areas of the cities in central and peripheral neighborhoods as well as different times of the day. Participants were between 21 and 60 years of age with education ranging from secondary school to university level. More than a half of interviewees had a university degree (in Helsinki, also many drivers in London were almost equally highly educated, while vocational training was typical for the drivers in St. Petersburg. In St Petersburg the average length of interview was 30 min, in Helsinki 37 min, and in London 52 min. Interviews in St Peterburg and in Helsinki were conducted in car with driver during the work-shift. Interviews in London were conducted over Zoom or another video-call platform (with or without video). The two first interviews in 2020 were made online, but due to difficulties in recruitment online, the rest with a research assistant in car later when covid-19 restrictions were removed. Drivers’ time was compensated with cash or gift cards with value of 15–40 euros (lower in Russia and highest in London and reflecting on the size of hourly earnings). Recruitment was easiest in Russia and hardest in London due to congestion charge, relative compensation level, and additional arrangements due to covid-19 for mediated interview. Interviews of St Petersburg drivers were conducted shortly before the global pandemic (9.12.2019 − 20.2.2020). Interviews in Helsinki (23.1.2020–02.06.2020) and London (10.11.2020, 24.11.2020–29.4.2021 − 16.6.2021) were conducted during the periods between the local spikes in pandemic. We were not aware of any systemic avoidance of interviews or difficulty in taking rides.

Thematic interviews were all recorded, transcribed, and translated to English (those done in Finnish or Russian). Interviews were coded and analysed by four different researchers in relation to thematic content. Consistency was gained through dialogue on interpretation, repeated analysis, and writing of the article with first author responsibility for coding and analysis of all interviews. In this article we report results from the 60 driver drivers working with platforms. Each driver was given information of the project and its purpose and all signed informed consent before the interview. The interview themes with Uber drivers included the following dimensions: working conditions; digital applications and surveillance; health, wellbeing, and social security; and the effect of covid-19 pandemic on work (Helsinki and London). The interview guide is provided as supplementary material in the annex of this article.

Our understanding of the regulatory context was based on the documents concerning legal cases, formal policy documents, and grey literature. We first examined relevant academic and policy literature, then compiled legal and policy documents through institutional and online sources and complemented this data with key informant interviews. Key informant interviews (26) focused on the regulatory context with trade unions (8), corporations (2), and where possible regulatory agencies at city, country (8) and European (4) level as well as with international organisations (4). Key informant interviews were made during 2021 (21.5.–08.10.2021 using online arrangement and recording (Zoom, Microsoft Teams)). Each participant received information sheet of the project prior of the interview and gave informed consent before the interview. Key informant interviews were used to complement, enrich, and locate policy documents and legal cases to provide a rich background for the assessment of the interview data as well as to understand better regulatory challenges and local contexts. This study reports on driver interviews.

Our research project has ethical approval 54/2020 from Tampere Region’s Humanities Ethics Committee, including for data management plan and archiving.

## Results

### City contexts

The initial market entry politics for platform companies were by 2019 over and Uber was present in all three cities. Uber had merged its operations in Russia and several neighbouring countries with those of Yandex already in 2017. In here we present Helsinki and London contexts shortly and the less discussed St Petersburg context in more detail.

In Helsinki in Finland, taxi reform in 2018 opened scope for Uber re-entry to the markets after a failed entry in the earlier strictly regulated legal context, and number of legal cases against Uber drivers [45, 48]. Investigative journalism reporting has shown that Uber influenced substantially how and on what ground taxi markets were liberalized in Finland [14, 48]. Addressing platform economy working conditions was also high on the political agenda as in 2019 Rinne/Marin government program included explicit focus on limiting scope for disguised employment [49].

In 1990–2013 there were between 9000 and 10 000 taxi licenses and taxi cars in Finland as each taxi car needed separate taxi license [50–51]. Taxi entrepreneurs formed half of drivers in Helsinki with rest of the drivers working as employees [50]. After liberalization of taxi market in July 2018 requirement of a separate taxi license for each car was removed and the number of taxi cars grew from 10 000 to 14 000 by September 2019 [52]. The number of taxi licenses peaked at the level 12 500 in 2020 and stabilized to 11 000 in 2021. If one has both licenses, it is possible to drive for own taxi company, taxi operator chain, or even be employed in a firm. Local taxi association and bigger taxi companies organize dispatching services to their taxi entrepreneur members. Eligibility to a particular dispatching service requires membership, but drivers can take rides also from Uber. Uber drivers operate predominantly under legislative framework for taxi drivers as entrepreneurs, 19/20 of interviewed Uber drivers were entrepreneurs. In Finland value added tax (VAT) is paid for income over 10.000 euros. Uber drivers pay 10% VAT for all income and a 25% commission to Uber. Drivers generally felt that they shared all the burden of VAT. Social security costs follow legislation for entrepreneurs, where obligations reside with driver-owners. Since 2018 Uber has provided a limited set of benefits in terms of insurance co-operation with AXA and a sum for parental pay [53]. In comparison to Finnish social security costs and coverage these remain low and follow more a global corporate policy. All drivers need to take an exam in either Finnish or Swedish (official language), but this no longer requires knowledge on local addresses. Finnish regulatory context is set both under EU internal markets and Nordic practices of social corporatism and collective bargaining. Regulatory authority on occupational health and safety is with Regional State Administrative Agency for Southern Finland, but since 2018 permissions have been under national Finnish Transport and Communications Agency (TRAFICOM) [54].

In St Petersburg in Russia the taxi sector is regulated by the several federal and municipal laws and government directives. The core of the regulation is Federal law from 21.04.2011 N69FZ, which represents a collection of amendments to multiple laws covering broad range of various aspects of transportation of people, including the work of taxi [55]. A new draft of a law on regulation of taxi sector was debated in the Duma for several years. Before 2022 Russian parliament was considering two alternative versions of the law. The more liberal version of the new law was drafted by the Government and its key innovations were about platform bases ride hailing services and strengthening to the role of digital services [56]. The deputies presented the alternative draft, and it mostly focused on strengthening the various modes of control over workers and taxi companies [57].

Ride hailing platforms take up to 32% the taxi sector in Russia and dominate in the big cities [58]. The rapid growth of Yandex.Taxi and other platforms have had a significant impact on reducing the share of the shadow economy in taxi sector. It is estimated that the share of shadow economy has reduced from 21% in 2015 to 12% in 2019. Uber/Yandex.Taxi is the largest “player” in Russian taxi sector and it controls over 40% of the taxi market [59]. Uber came to Russia 2014 mostly focusing on individual drivers but already in 2018 Uber merged its operation with Yandex (in a similar manner it left China, and Southeast Asia markets) unable to compete with large local IT company. The Uber app gradually retired in Russian market. In 2021 Uber owned 29% stake in a joint venture with Yandex.Taxi, Yandex drivers continued broadly using Uber brand, while the actual operation of the platform was managed by Yandex.Taxi. Amid Russian military aggression against Ukraine in 2022 Uber announced plans of selling the remining share in a joint venture with Yandex [60]. Currently platform companies share their data related to taxi rides with local transportation authorities. A new law gives security services an “automated remote access” to platform companies’ databases [61], on the other hand, the law introduces a set of measures aimed at increasing the safety of taxi work.

In Russia government has sought to push drivers to register as entrepreneurs with incentives, but at the time of this study most drivers operated as “partners” registered as drivers at taxi fleet companies (taxi parks), which may also lease cars or simply act in the role of mediating legal entity between a driver and a platform. We may assume that the majority of 600 000 taxi drivers in Russia aren’t employees of taxi parks, nor they are self-employed [58]. Their status is “in-between” employee and entrepreneur, and the recently adopted legislation formalized this arrangement.

The taxi fleet companies offer drivers a range of services: registration as “partner”, rent of the car, rarely employment. In 2022 a lease of Economy-class car would cost approximately 1500–2500 rbl /day (approximately $21-35) with obligation for driver to work 6–7 days a week. Taxi fleet companies are responsible for the maintenance of cars. Even if interviewed drivers often assumed that their “salary” was paid by the platform, they were not in employment relation with a taxi fleet company. Taxi fleet companies deduct taxes directly for the payments from the app as well as a commission, in 2020 25%.

Yandex.taxi was actively promoting the model of direct partnerships with self-employed drivers offering them bonus points and services such as extended insurance. Yandex.Taxi automatically applies accident insurance policy for drivers from the moment they accept the assignment to the moment of completing it. The driver can’t receive an insurance payment if he or she grossly violated traffic rules [62]. Self-employed drivers who are partners of the platform and have a high rank in the reward have access to free of charge extended insurance policy including: sick pay on the starting from the third day of sickness (including COVID-19) and insurance for time online waiting for assignment [63]. In 2022 the sick pay was 2000 rubles (approximately $28) per day.

London is a relevant location for Uber and substantial source of revenue for the company. UberX initially gained a license to operate already in 2012 with a view to ensure service for the Summer Olympic Games hosted in London [64]. However, Uber has later been struggling with local regulator Transport for London, which declined Uber license due to concerns for unregistered drivers in 2017 and 2019 [65–66]. A supreme court case on worker status took place in February 2021 [67]. In contrast to employment, the worker status sits between self-employment and employment providing drivers with certain benefits, such as holiday pay and minimum wage guarantee. Uber also struck a deal with a trade union GMB to represent its 70.000 drivers in 2021 [68]. In 2022 Transport for London approved again the Uber operator license [69–70].

In London three types of taxis operate under specific licenses, London black cabs, private hire cars and platforms, such as Uber and Estonian Bolt. Uber (67%) and Bolt (21%) are leading platforms in ride-hailing [71]. They operate formally as private hire cars, but they can operate in a wider geographical area and under different set of specific regulatory requirements.

In London Uber court case was reflected in driver interviews as it resulted in tangible benefits for drivers who could claim holiday pay and other benefits as result of “worker” status [68, 72]. In the UK two trade unions, GMB and ADCU -unions, represent Uber drivers.

International regulatory context has become more challenging to global corporations. Uber has now declared it is moving out from Russia and during the project United Kingdom has moved out from European Union. However, key court decisions still matter. This is the case for the UK supreme court for the Commonwealth countries and for European Court of Justice for EU Member States. Court cases act as trigger to court cases in other countries. While these are decided in national context, the landmark cases give precedence and scope to further cases and decisions. This implies that while global platform economy corporations may have sought to disrupt, they have eventually complied with court cases and decisions by authorities such as Transport for London. In this respect the governments and regional or city regulators will have the essential role in guiding of how and on what ground platform companies operate now and in future.

In all cities there were common concerns by drivers with grievances towards high level of commission by Uber in comparison to other providers. In all cities drivers used more than one platform or taxi/private hire for driving opportunities. Here we report main findings arising from reading and coding of the driver interviews and relating these to the working and employment context in each city.

In Helsinki Uber entry to the taxi market coincided with a shift from a regulated relatively well-paid profession to an easy access low pay profession, with a variable service quality during the transition. In Saint Petersburg taxi use has increased along platformisation but working conditions of the taxi drivers have changed in ambiguous ways. On the one hand, people use taxis as a means of public transport, so work is available, but the work intensity has increased because low wages need to be compensated with longer working hours. On the other hand, platforms offer drivers more sense of safety, as identified customers are registered in the app that the drivers are working with. In London Uber presence has been longer with flexibility in working-time with more competition, but contested practice as well as gains for drivers as result of court cases.

### Compensating incomes with working hours

A key finding for health and wellbeing was that driving Uber required to compensate low pay and higher costs by longer working days. While in the UK and Finland the limit for driving has been set to 12 h, in Russia this was 16. The key mechanism to maintain income was to drive more hours. Concern over income vs. time use was articulated in all three cities:

*‘Uber drop their prices. For example before if I was making hundred pound a day working 10–12 hours now instead I make like 70 or 80 because of this drop in the prices. They should raise their prices back. The fare. The fare is very low. Uber I think has the lowest which is not good for us. It’s affecting all the drivers. You need to work long hours and you’ll be tired. And also health wise is no good as I said. These are all negative. You always sitting here. You’re not active. I hope I answered all your questions*. (L16)’ (London).

*‘I have friends who, drive Lähitaksi and so on, and we were talking about it, when was it, well it doesn’t matter, we have almost the same turnover. But the only difference is I have to drive twice as much as him because he has different prices [chuckles]. I can tell you about work, anonymously, Uber calculates in a pretty detailed way how long the car has been in traffic (- not just traffic), if it’s a normal shift it’s ten hours, so I drive (like a robot) okay, I can drive, I drive for ten hours. Okay I have stops but you try driving for ten hours [laughs*]. (H03)’(Helsinki).

*‘Yes, I told you everything is changed. I just gave an example of what happened before… About 5 years ago, I could use my car for 3 weekends to earn 30,000 rubles, and that’s it; and I can forget about it for a week. And here now in these aggregators (term used for taxi platforms such as Uber or Yandex), that is, it is impossible to do this. This is possible, but, firstly, Yandex gives 16 h of driving, then you need to rest. Well, this is understandable. Well, there are narrow opportunities with these aggregators, unrealistically narrow. Therefore, you can’t jump above your head here. Without these aggregators, everything was fine, and you could jump above your head.* (SP05)*’*(St Petersburg).

In all three cities Uber drivers were struggling to meet costs of car maintenance with income from driving and the relatively high commission by platform. The low income and lack of possibilities to set prices through other means than driving was evident. This was brought up especially by drivers who had invested in a car for better income as this was difficult to gain back by driving only.

While platform corporations have emphasized the benefits of flexibility of work, it is clear, that many platform workers do work fulltime and seek to compensate lower rewards through longer working hours. Long working hours were particularly prominent in St Petersburg, while in Helsinki and London driving Uber was also used as a flexible option.

### Regulation and autonomy

In all cities informalization and easier entry to markets has become reflected in regulatory incentives or measures to curb grey practices and subcontracting. Informal work was at the core of cases with respect to banning Uber from practice in London [65–66]. In Finland driving taxi without appropriate license was important in initial phase of Uber arrival to Finland and legal action against Uber. In Russia informality is reflected in requirements for verification of driver status, but as well frustration on limits and lack of platform workers interests to do so. To typify the liberal UK regulatory context resulted in highly contested legal measures both on behalf of drivers and regulators (London Transport Authority), whereas in Finland de-regulation strengthened the role of Uber drivers working as entrepreneurs in wider less regulated taxi markets. In Russia platforms provided potential means to regulate wild markets, yet driver preference was more geared in gaining distance from both government and regulatory efforts of platforms, and taxi parks proved a useful “firewall” for this. The firewall thus functions as a two-way insulation leaving drivers invisible and away from the government.

In all cities flexibility of when to drive was appreciated and drivers felt the app was competent. Attitudes towards surveillance varied. In Russia the app followed driving performance and speeding could lead to disconnection, which was felt as unfair. Drivers seemed in practice less concerned of surveillance than they were of unfair disconnection or rating. Concern over disconnection was of less concern in Finland in comparison to St Petersburg and London. This could be due to number of reasons, such as a viable alternative option of operating successfully as an independent taxi-driver-entrepreneur.

The different rating levels for algorithmic management were recognized, but only few admitted that they paid attention to keep up with the different levels. Keeping up with the level was an expressed source of stress or reason for driving long hours, but coping with costs, getting enough rides, and being disconnected were more directly relevant cause of stress. The role of disconnection was brought up in all cities, but most in London. This may be due to a court case with Uber [73], but it was raised also in other countries. Uber was felt to dismiss drivers too easily without giving a chance to being heard when customers gave complaints or poor reviews of drivers. Having a long history as an Uber driver means you have earned a significant trust capital to your app profile. The one-sided ability to disconnect gives the platform company powerful position to decide (or at least a potential for it) on the future of your profile.

### Surveillance and safety

The surveillance of drivers (tracking location, speed), identification of customers through their credit card registered to the system, and lack of cash in the car due to secured credit card payment were all seen to add to safety of the drivers. Angry and drunken passengers were a key safety concern. The mutual rating system in contrast to driver assessment only was appreciated as drivers could indicate problems with customers. Drivers felt that not being able to see the destination was a concern as they felt forced to blindly accept the rides without knowing the destinations. While online support was available, this did not necessarily result in action. The response times for driver verification and concerns were raised in St Petersburg and London, while in Finland online assistance was appreciated. This may relate to the higher volume of drivers in London and St Petersburg. The calculation of time and distance was concern as traffic congestion or long-distance driving to pick up the customer were not considered in the payment. There were mixed views on different aspects of surveillance, the focus on speeding was more prominent in St Petersburg, but not in London or Helsinki. The surveillance as such was not always considered as a negative issue.

*‘I: But did they punish drivers for speeding or something? L06: No. They have never punished me but it doesn’t make sense. You don’t sit on an app and monitor somebody’s speeding. London is a busy city. We have too many cameras here. If I speed I have a responsibility of my own license. I will get ticket. That’s not a business. So long the passenger is safe I’m a driver. My responsibility to drive responsible. To transport the passenger to a (safety) area. You are expect me to speed with the passenger. I’ll be stupid to do that.*

*I: But is there any aspect of monitoring that worries you or makes you?.L06: No. I don’t think. No. Because if you put the apps on it tell you they will notify where you at. They have to because they have to look for customer for you. Therefore it don’t bother me. You can look for me. I don’t care.*’ (London).

*‘Uber gives us security. Like before you register with your name. At least your address before you can order Uber and then it’s taken from your credit card directly. Such that even if you want to place, even if there’s some disagreement or problems they can be able to identify you. Uber actually has some degree of security for the driver by knowing who the driver is carrying. It’s also a good customer service for me because if I know you are Matti and I just arrive I say good day Matti. That’s really impressive. Sometimes you go pick a Yango rider. Hello. You don’t know. Of course you don’t know his name. It’s not it. (H02)’* (Helsinki).

*‘It (surveillance and fatigue tests) is also a positive one. This is not a negative. Yandex is a good app, but I haven’t seen its disadvantages yet. That’s right, 16 h a person if. If something happens to the passenger, it is very difficult, on both sides. Both in this life and the next life, it will be very difficult for a person. (SP19)’*(St Petersburg).

### Economic and social costs of Uber driving

In each city many drivers were also familiar with key cost issues. The most dissatisfied were those who had invested in driving with clear calculations on benefits and costs of different options just to make the ends meet. Once Uber enters to new markets like in 2018 to Helsinki, it attracts new drivers with charging a lower service fee, but it ends to the level of 25% + VAT, which was commonly regarded as a high commission. There was frustration to the promotions that new drivers receive that may have given a rosier picture of the earnings. Those with existing asset (car) were in a less vulnerable position than those who had to pay for a loan or renting of car. In Finland difficulties to find other work as educated immigrants came up more clearly, whereas in the United Kingdom drivers had a moved from other professions. In Russia immigration was from post-Soviet states.

The understanding of social security or insurance issues differed among drivers and were more known for those with longer time driving in taxi industry. As most of those interviewed were immigrants, their concerns were less concerned with social protection. In Finland most Uber drivers were entrepreneurs, who pay their social security themselves. Many were not fully aware what kind of insurance payments they should pay and what kind of social protection they are entitled. In London, the worker position (the 3rd category of work) that supreme court ruled to Uber drivers was recognized with the holiday pay taken up by drivers [72]. The Russian drivers were in a weaker position in relation to social security with avoidance of contact to authority and payments. Social security was thus based on informality in Saint Petersburg with drivers reliant on basic health insurance provided by state and municipality and aware that in case of accident they will have to cover themselves the costs of medical care. In Finland and UK this was not an issue due to residence-based access to adequate health care.

In all cities the role of informal markets and Uber driving as choice for migrants was present either directly or indirectly. This increases the power imbalance between drivers and platforms and is likely to increase vulnerability. Racism or ethnic discrimination either in covert or explicit reference was taken up more in Helsinki than in London or St Petersburg.

Drivers in the three cities were older and few did other “gig-work” indicating that asset driven jobs such as Uber might be in practice at the “top of the line” of on-location platform work. This would fit to the arguments on hierarchies between platforms [21], even if Uber would not be “top of the line” in the USA. Being a professional driver requires resources and capabilities, such as access to credit/capital and sufficient knowledge of local language to get licenses. This may be contrasted to lower threshold on language and starting capabilities and investment in access to food delivery work.

### Disbalance of power: tradeoffs

Finally, while platform companies address drivers as partners, this was not a shared feeling as most drivers in all cities implied a more one-directional relationship. While the drivers were able to work for different platform companies or independently, once they worked for Uber or Yandex, tasks set were defined by corporations alone. Platform companies gathering of data and surveillance of drivers was not as much an issue as was the lack of support when problems emerged or with delays in response.

*‘I: How do you see these rating systems or algorithms, allocating clients or drives.? L13: You know what (what the Uber have said) that the algorithm is based whoever is the closest to the job, oh well, I don’t know about that. I don’t know if I can trust them or not you know what I mean but, the thing is as well that for example, I have a job two miles away so I’m thinking like oh there must be somebody closer, but perhaps, the other drivers rejecting the jobs so then it goes to another closest one, so, yeah and they’re rejecting the jobs because again it’s either central London or it’s just not paid that well. And the ratings, oh well with the rating on the end of the day the customer can say whatever he wants. So, even when you are a good driver he can still give you a one-star, just because he felt like it.’* (London).

*‘I: What is it like, do you get help at the Uber office? Do they help you drivers? H01: They’ve always helped me, but they open only at 11 or something, and it’s closed weekends. My friend just told me that the application was saying that a new photo should be sent, well he tried to take the photo in the car and you could only see half his face, the device froze, and it wouldn’t accept anything so he had to wait until Monday morning 11 o’clock to be able to turn on the device*. (Helsinki)

*I: So if something like this happens in the weekend? H01: Yeah because it gives you all these notifications like send a new picture. For me too it sometimes tells me to choose a vehicle and such, I don’t know, even though I don’t do anything about it, why these happen. It has a life of its own.’* (Helsinki).

*‘SP09: Yes, they make it worse and worse every time. Recently one guy, every 10 days… the photo control In Yandex app, and you send reports. He takes 6 photos and sends them. Sometimes you wait for 2 h for this data to be confirmed or not confirmed. Sometimes you wait an hour, two, three, four. You can’t take any orders, nothing. Here you sent photos and wait for this stupid Yandex to send you confirmation. Either accepts or does not accept. Irritate. I had a situation once, I went from 9 o’clock, took a picture of the car, sent it. And only after lunch at 4 o’clock I received confirmation. Can you imagine.’* (St Petersburg).

While Uber and Yandex.Taxi treat drivers as “independent contractors” or users of their “information services”, drivers experience intensive algorithmic control more as pronounced disbalance of power. A driver may lose access to work for several hours, days or permanently because of “low activity”, “low rating points”, problems in automatic authentication — issues where a driver has limited control.

## Discussion

Our results resonate with studies on health and social implications of platform work [1–8, 74–75], but they also provide argument for improved regulatory oversight. Our findings also fit with the practice of “contentious compliance”, where firms comply with existing rules to expand, while seeking to deregulate or change them for their benefit [76]. During the time of interviews (2021) European Union directive proposal on working conditions [78] and guidance [78] on competition law and collective agreements raised expectations for improved regulatory oversight, however its impact is likely to be limited [79–80]. Our results indicate the importance of the existing regulatory framework in shaping of the regulatory context of platform work. In London Uber started with a license but ended up challenged by regulatory authority and court cases in line with wider regulatory context [64]. The initial start in Finland was disruptive [15, 17, 48] and the change of taxi law can be seen as a window of opportunity for Uber to emerge in Finland, but the operational context also reflects the wider existing regulatory framework for taxi drivers as entrepreneurs. On the other hand, in Russia where Yandex has benefited from less regulation of the employment in the taxi sector, the shadow worker qualities have continued. Platform operators may bring about and benefit from disruptive change, but as firms they will adjust to different regulatory, employment and operational environments and culture. Furthermore, platform providers gather data, which could be used to open scope for strengthening social protection in informal work more globally.

Our results are in line with Benach et al. [35], emphasis of platform economy as a social determinant of health as well as research emphasizing importance of terms of employment [81–82]. The direct impacts of platform work upon health and working conditions may depend on type of work, nature of algorithmic management, and operational priorities of the platform. For ride hailing self-reported psychosocial impacts from stress and algorithmic surveillance and rating were more limited than we expected on the ground of wider concerns on surveillance and rankings [22–8]. In contrast, oversight was seen by some as an element for security by identifying the customer and pre-secured credit-card pay. This as well as driver disappointment in support from Uber and feeling of Uber being always on the side of customer resonates with study on platform workers and job quality in Scotland [29]. Stress and dissatisfaction were more clearly articulated in the context of terms of and basis for disconnection. Drivers have little control when at work or conditions of dismissal, while the low pay may not fully compensate for efforts leading to added stress to gain enough rides to compensate the high commission share and increased costs for the driver. The diminishing or limited rewards from driving required driving overtime to gain income. These elements were also mentioned by drivers in all three cities. In this respect our results echo other studies on on-location platform work [1, 4–10, 22, 24, 29], while in Poland working with Uber required further action beyond mere increase of working hours [27].

The drivers in Helsinki and London were slightly older and more educated than in St Petersburg. It is possible that more educated drivers were more willing to participate in the study, but if this would have been the case, it should have been more present in London, where proportionally more drivers declined interview. In each city there was internal variation in responses and driver backgrounds to suggest that our sampling was not biased beyond what can be expected from the method. We assume that any fully informal or illegal drivers would not have participated in any case. Most of the drivers were men and of immigrant status, which reflects earlier general findings in on-location platform work.

While Uber kept some aspects of operation the same across countries, it has been able to adjust its actions between countries to fit to the local legislative context. Court cases, research, and investigative journalism would imply that platform economy providers also seek to shape the legislative context at European level [81–84]. Our research supports findings that national and regional legal regulatory frameworks remain important for working conditions of platform workers [84]. Our results also fit well with findings by Thelen [44] comparing Uber in US and European Union and in pointing out the importance of the business model of platform corporations for how they operate in different country contexts.

It is important that when working conditions of platform work are addressed this is not done narrowly or merely in terms of occupational health and safety conditions and environment, but also in terms of conditions of employment, transparency, and how algorithmic management is used. Platformisation is a social determinant of health even if the actual work may not imply high risks or poorer working conditions in comparison to other “like” work for taxis, bus, or delivery drivers. The driver concerns were with declining terms of work and one-sided power to “disconnect” or a threat of disconnection, building an invisible unbalanced power structure between platform companies and workers. At the core are thus employment conditions and power relations.

As drivers face increasing costs with limited means to increase income beyond longer working hours, this is likely to frame further discussions on platform work. One of the defining features of working with Uber has been the lack of capacity to higher prices, when costs increase. Employment status and regulatory context are also related to the scope of unionization and court cases as has been the case in United Kingdom and Spain [85–86].

Working hours do not usually calculate waiting or pick up times so the gross hourly pay remains lower than calculated on the ground of effective working hours on the app. The switch between different platform applications may also lead to extra working hours in total even if they would not exceed 12 h limit for one provider. This is a health and safety issue. An effective occupational safety control would require a joint presence of platform for all apps in relation to working hours. While this could be technically feasible it is likely to gain opposition especially as drivers were compensating poor income with excessive hours. As platform companies collect the data, they could provide an avenue for local and national regulators to enforce and enlarge obligations for social protection as well as for health and safety protection.

## Conclusions

Our results bring added value to the importance of national and local context, regulators, and law to global platform corporations as well as a more mixed perspective in relation to implications from algorithmic surveillance and monitoring. They also indicate importance of terms of employment for platform work, but also locate a health and safety concern as result of increasing working hours by shifting from one app to another especially when prices are set and increasing working hours remains the only means to enhance income.

It is important that when working conditions of platform work are addressed this is not done narrowly or merely in terms of occupational health and working environment, but as well in terms of conditions of employment. While rating systems and algorithmic management have been seen as potential concern, in our study drivers saw these as less problematic than one-way or unpredictable disconnections or sense of not being valued by corporations prioritizing customer views and priorities. Data gathering practices by platform companies indicate that national and local governments have scope for improved social protection and health and safety for platform workers.

In terms of disruptive impacts of platform corporations our results indicate that while corporations may seek to change the regulatory frameworks, they do comply with the legal requirements set in each country or locality. In London changes resulted from legal cases. The high commission margin, pricing practices, and uncertainty with disconnection were raised by drivers as issues of concern. In contrast, drivers felt that surveillance and lack of cash in the car improved safety.

Our results are in line with wider concerns on platform work, precarity, and the importance of power inequalities in platform work. However, they also highlight the importance of national and European level regulatory frameworks and their relevance for employment conditions. The lack of control of terms of work, apart from the autonomy concerning when to work, as well as bearing all risks and costs related to employment was reflected in interviews of drivers in all cities, even if measures to address this differed between cities and local regulatory contexts.

### Electronic supplementary material

Below is the link to the electronic supplementary material.

Supplementary material 1: Annex: Interview guide

## Data Availability

Our interview material will be provided anonymized and in accordance with permission of those interviewed to the Finnish Social Science Data Archive after analysis and can be provided upon request as an Atlas.ti file. Documentary data is based on publicly available sources. Our interview guide is in the annex as supplementary material.

## Declarations

### Financing

This research was funded by Research Council of Finland grant 325253.

## Abbreviations

VAT: Value added tax
