# Supplementary material for: Globalization, platform work, and wellbeing—a comparative study of Uber drivers in three cities: London, Helsinki, and St Petersburg
Source: Global Health. 2024 Mar 1;20:18. doi: 10.1186/s12992-024-01021-3 (PMC10908096; doi:10.1186/s12992-024-01021-3)
Supplement: Supplementary file 1 — Supplementary material 1: Annex: Interview guide [file 12992_2024_1021_MOESM1_ESM.pdf]

Interview guide. Created 3.12.2019, edited on 25.05.2020

**Kuljettajat**<br><br>**Taustatiedot**<br><br>Sukupuoli<br>Ikä<br><br>Siviilisääty<br>Koulutus<br><br><br><br>Asutteko täällä PK seudulla, kuinka pitkään olette asuneet täällä? Entä sitä ennen?<br><br><br>Mitä kieliä käytät?<br><br>Missä kategoriassa ajatte (UberX, Black)<br><br>(Kuljettajana toimimisen tapa suhteessa Uber/Yangoon)<br><br><br><br>Onko taksi teidän päätoimi?<br><br>**Voimmeko sinutella?**	**Водители**<br><br>**Общая информация**<br><br>Пол (не задавать)<br>Возраст<br><br>Какое ваше семейное положение?<br>Какое ваше образование? (одно или несколько, работали ли вы по той специальности на которую учились?)<br><br>Вы живете в Питере? Как давно? - Где жили раньше?<br><br>В какой категории такси вы работаете (Эконом, Комфорт, Бизнес…)<br><br><br>Как вы оформлены в Яндекс-такси? Через парк или напрямую как индивидуальный предприниматель (ИП), самозанятый? Почему?<br><br>(Это ваша основная работа?)	**Drivers**<br><br>**Background information**<br><br>Gender<br>Age<br><br>What are your family relations/are you married?<br>What is your professional education? (one or several? Did/do you work in your profession?)<br><br>How long have you lived in this city? Where did you live previously?<br><br>Which languages do you use?<br><br>In what category do you drive (UberX, Black,...)<br>(questions about contract with UBER, via park, direct partner-entrepreneur or freelancer)<br><br>Is driving Uber your main job?
**Taustoja ja kokemus Uber/Yandex työoloista**<br><br>Kuinka pitkään olet ajaneet taksia?<br><br>Miten päädyit ammattiin?	**Условия работы в (Яндекс)такси**<br><br>Как давно вы работаете в такси?<br><br>Как вы пришли в эту профессию?	**Work conditions in Uber**<br><br>How long have you worked with Uber?<br><br>How did you come to this profession?

Kuinka paljon tunteja ajat tavallisena päivänä / viikkona? Mikä applikaatiota käytät?<br><br>Mitä kautta saat tilauksia? MItä taksialan toimijoita käytät asiakkaiden hankinnassa?<br><br>Mitä ajattelet Uberilla ajamisesta?<br><br>Mitkä asiat Uberilla ajamisessa on hyvää?<br>Mitä haluaisit muuttaa Uber-työssäsi?<br><br>Onko teillä kanta-asiakkaita, joita palvelette keskinäisen sopimuksen pohjalta?	Сколько часов в день вы обычно работаете в такси? В неделю? С какими агрегаторами и парками вы работаете?<br>Расскажите, что вы думаете о работе в Яндекс-такси?<br><br>Какие вы видите положительные стороны в работе с агрегатором? (вам нравится работа в такси с агрегатором? Что именно вам нравится/не нравится?)<br><br>Что вы хотели бы изменить?<br><br>У вас есть постоянные клиенты, которых вы возите отдельно от работы с агрегатором?	How many hours do you work in a day/week? Which apps and companies do you work with?<br><br>What could you say about driving with Uber?<br><br>What positive aspects do you see in driving with Uber?<br><br>What would you like to be different (in driving with Uber)?
**Applikaatio**<br>Mitä ajattelet Uberin/Yango applikaatiosta?<br><br>Mitkä siinä on hyvää?<br>Mitä haluaisit muuttaa applikaatiossa?<br>Miksi? Voitko vertailla muihin asiakashankinnan applikaatioihin (sis. myös perinteisen asiakasvälityksen)? Mikä on sinulle tärkeimmät erot?<br><br>Mikä vaikuttaa siihen, miten saat kyytejä applikaation kautta?<br><br>Mitä ajattelet Uberin/Yangon asiakaspalautejärjestelmästä?<br><br>Mitä vaikutusta asiakaspalautejärjestelmällä on työhösi? Mikä on sinun tähtitasosi?	**Приложение**<br>Что вы думаете по поводу приложения Яндекс?<br><br>Какие плюсы?<br>Что вы хотели бы изменить в нем?<br>Почему?<br>Вы могли бы сравнить с приложениями других служб, в чем важнейшие для вас отличия?<br><br>Что воляет на получение заказов (уровни активности, рейтинг, корона)? Какая у вас средняя оценка клиентами? А какой уровень активности? Есть ли какие либо приоритеты (бронза, серебро, платина, наличие короны)<br><br>Что вы думаете о системе клиентских рейтингов в приложении Яндекса? Как они влияют на вашу работу?	**The App**<br>What do you think about the Uber application?<br><br>What's good about it?<br>What would you like to change in the application?<br>Why? If you compare to other apps, what are the most important differences?<br><br>What has influence on how rides are distributed between drivers?<br><br>What do you think about Uber’s customer feedback system?<br><br>What impact does the customer feedback system have on your work?

Mikä on pistetasosi (30-100; jos järjestelmä on Suomessa käytössä)?<br><br>Mitä etua korkea taso antaa sinulle (tieto määränpäästä ja olet priorisoitu kuljettaja)?<br><br>Mitä ajattelet siitä, että palaute- ja pisteytysjärjestelmät tulisivat laajemmin käyttöön koko taksialalla?<br><br>Mitä ajattelet applikaation kautta toteutettavasta mahdollisesta kuljettajan valvonnasta? (Nyt puhutaan paljon sitä että jatkossa sovellus valvoo kuljettajia tehokkaammin, mitä mieltä olet tästä?)<br><br>Tekninen: ylinopeus, ajotyyli<br><br>Kuljettajan kunto mm. väsymyksen seuranta<br><br>Miten koet työssä, kuinka paljon sinulla on vaikutusmahdollisuuksia työsi organisointiin?<br><br>10 erittäin paljon, 0 järjestelmät tai muut päättävät puolestani<br><br>Mitä vaikutusta on sopimusehdoilla Uberin, Yangon tai TaksiHelsingin kanssa; ehtoja tai vaatimuksia työvuoroihin tai - tunteihin liittyen?<br><br>Mitä applikaatiota käytät osana asiakashankintaan (Uber/Yango)? Käytätkö kuljettajana muita applikaatioita?	Сейчас есть дискуссии о том, что в дальнейшем приложение больше следить за водителем?<br><br>Техническое наблюдение: превышение скорости, стиль вождения<br><br>Наблюдение за физическим состоянием водителя: усталость.<br><br>Как бы вы оценили насколько у вас есть возможность влиять на то, как вы организовываете свою работу (или рабочий день)?<br>10- влияю очень сильно<br>0 - приложение и другие влияют очень сильно<br><br>Расскажите подробнее? (Условия договора с таксопарком?)<br><br>В своей работе какие приложения вы используете? (какие приложения у вас сейчас включены в телефоне? Приложения для такси, карты, заправки, другое)	What is your level in the app (silver, gold, platinum)? How many points do you have?<br><br>What priorities/benefits do they give to you?<br><br>What do you think about driver monitoring through the application? (There's a lot of talk about how the app will monitor drivers more intensively, what do you think about that?): speeding, driving style, driver's condition fatigue monitoring.<br><br>How do you feel at work, how much influence do you have on how you organize your work?<br><br>10 very much, 0 the app or others decide for me.<br><br>What does this mean? (terms of contract with Uber or taxi park)<br><br>Are you using an application other than Uber / Yandex?<br>• When do you use something else?

**Työn luonne ja vastuunjako**	**Характер работы и разделение ответственности**	**Nature of work and division of responsibilities**
Mistä Uber ottaa vastuun?<br><br>Onko Uber työnantaja?<br><br>Mistä odotat Uber/Yangon vastaavan?<br><br>Mistä sinä itse kuljettajana koet olevasi vastuussa?<br><br>Kuka päättää työpäivän pituudesta, työvuoroista, missä ajaa? (siltä osin kun ei ole aiemmin keskusteltu)<br><br>Perutko tarjottuja kyytejä (tilauksia/keikkoja) ?<br><br>Jos perut, mitä siitä seuraa?<br><br>Kuka hoitaa verot ja eläkemaksusi?<br><br>Kuvaile normaalityöpäivääsi (Uber/Yango)(?)<br><br>Kun työssä jokin ei toimi, kenen puoleen käännyt<br><br>- taksifirman<br><br>-Uber/Yangon<br><br>- kollegan<br><br>-ammattiliiton<br><br>Minkälaisia jännitteitä on Uber/Yangon ja muiden taksien välillä? (myös maahanmuuttajakuljettajien ja paikallisten välillä)<br><br>Mikä oli viimeisin viesti mitä sait Uberilta/mikä oli aihe?	На ваш взгляд, за что отвечает таксопарк/обязанности таксопарка? (если работает с таксопарком) За что должен отвечать таксопарк?<br><br>За что отвечает Яндекс (обязанности Яндекса)? За что должен отвечать Яндекс?<br><br>По вашему мнению, каков у ваш круг обязанностей как водителя? (за что вы отвечаете как водитель)<br><br>Кто принимает решение о продолжительности рабочего дня, когда выходить на линию, где ездить?<br><br>Вы знаете, как оплачиваются налоги с вашей зарплаты? А пенсионный сбор? Другие обязательные выплаты (страховка, медицинское обслуживание, ТО)<br><br>Расскажите о вашем обычном рабочем дне в такси (Яндексом)?<br><br>Если в работе возникают какие-то сложности/ трудности/проблемы, к кому вы обращаетесь<br><br>а) в таксопарк<br>б) в яндекс/агрегатор<br>в) к коллегам<br>г) профсоюз	What Uber is responsible for?<br><br>Is Uber an employer?<br><br>What do you expect Uber to be responsible for?<br><br>What do you, as the driver, feel responsible for?<br><br>Who decides about the length of working day, where to work, where to drive?<br><br>Are you canceling the rides offered (orders)?<br><br>If you cancel, what will happen?<br><br>Who manages your taxes, pension and other obligatory payments?<br><br>Describe your normal day of taxi driver (at Uber)(?)<br><br>When something does not work, to whom you turn to<br><br>(a) a brokerage firm<br><br>b) Yandex<br><br>c) a colleague<br><br>(d) trade unions

Tuliko se applikaation kautta vai sähköpostiin?<br><br>Muista viimeisen kerran kun otit yhteyttä Uberiin, mikä oli asia? Saitko sen selvitetty?	Какие есть напряженности в отношениях между<br>• Агрегаторами<br>• Между водителями и агрегаторами и парками<br>• Между водителями (включая этнические напряженности)	What are the tensions between Uber and other taxis? (also between migrant drivers and locals)<br><br>What was the last message you got from Uber? Did it come via app or to mail?<br><br>When did you contact Uber last time, what was the issue? Did the issue get solved?
**Korona**<br><br>Miten Korona epidemia on vaikuttanut sinun työhösi?<br><br>VAIHTOEHTOISESTI<br><br>Miten taksinkuljettajalla /ruokalähetillä menee näinä Korona-aikoina sinun näkökulmastasi?<br><br>Mistä olet saanut Koronaan liittyvää terveysturvallisuuden ohjeistusta epidemian aikana? (mitä kautta)<br><br>Minkälaista ohjeistusta olet saanut?<br><br>Mitä uusia käytäntöjä olet ottanut käyttöön Koronan takia?<br><br>Vain taksit: Oletko siirtänyt työn painopistettä ihmisten kuljettamisesta esim. ostosten tai tavaroiden kuljettamiseen? (Ruokakassit;		How has the Corona epidemic affected your work?<br><br>ALTERNATIVELY<br><br>How does a taxi driver / food courier manage these Corona times from your perspective?<br><br>Where did you get your Corona related health and safety guidance during the Corona epidemic? (through which channels)<br>What kind of guidance have you received?<br>What are the new practices you have applied because of Corona?<br>Taxi-only: Have you shifted the focus of your work from transporting people to, for example, shopping or transporting goods? (Food bags; additional questions if significant)

lisäkysymykset jos tätä ollut merkittävästi)<br><br>Minkälaisia terveysriskejä tunnistat asiakkaan ja kuskin/lähetin (ja auton) kohtaamisissa Korona-epidemian aikana?<br><br>Taksi vain: Mitä eri välineitä (esim. maksit tai pleksit) näet keinoina vaikuttaa Koronan tarttumisriskiin työssäsi?<br><br>Taksi vain: Oletko tiennyt kuljettavasi Korona-tartunnan saaneita? Jos kyllä, miltä se on tuntunut? (täsmennä: sairaalaan / terveyskeskukseen viennit)<br><br>Minkälaista stressiä tai henkistä kuormitusta Korona-aika on sinulle aiheuttanut? (tartuntoihin liittyvä tai talouteen liittyvä)<br><br>Miten Korona epidemia on vaikuttanut työtehtäviesi (kyytien/kuljetusten) määrän ja ansioihin? Prosentteina.<br><br>Oletko pitänyt taukoa työnteossa Korona-aikana?<br><br>Kysymme Koronaan liittyvistä tukimuodoista.<br><br>Oletko joutunut Koronan vuoksi karanteeniin? Oletko hakenut tartuntatautipäivärahaa?<br><br>Oletko hakenut Suomen hallituksen Koronan aiheuttamiin vaikeuksiin tarkoitettuja tukia?		What health risks do you identify in customer-driver / courier (and car) encounters during the Corona epidemic?<br><br>Taxi only: What different tools (e.g. mask or plexiglass) do you see as ways to influence the risk of Corona influences in your work?<br>Taxi-only: Did you know you were transporting Corona-infected people? If yes, how do you feel about it? (To hospital or Corona cliniques)<br>What kind of mental stress has Corona caused you? (Stress on infections or on economic consequences)<br>How has the Corona epidemic affected the number of your work tasks (rides / transports) and earnings? In percentages.<br>Have you taken a break from work during Corona?<br><br>Now questions on Corona related social support programmes.<br>Have you been quarantined because of Corona? Have you applied for an infectious disease daily allowance?<br>Have you applied for grants from the Finnish government for the

Mitä ajattelet yrittäjien Uber-kuljettajien sosiaaliturvasta esimerkiksi työn vähenemisestä johtuvan tulotason pudotuksen korvaamisesta?<br><br>Oletko hakenut tukea (emoyhtiöltäsi) Uberilta/Foodoralta/Woltilta Koronan vuoksi esim. sairauslomat, maskit tai desinfiointiaineet?<br><br>Miten koet eri tukimuodot, jotka on tarkoitettu Koronan aiheuttamiin vaikeuksiin?<br><br>Tunnetko Uber-kuskeja (Foodora/Wolt-lähettejä) joilla on ollut tartunta tai heidät on määrätty karanteeniin?<br><br>Miten he ovat kertoneet sairaudestaan tai karanteenistaan muille ja Uberille?<br><br>Kuinka yleistä on että ihmiset jättävät kertomatta näistä? Tiedätkö näin tapahtuneen?<br><br>Mitä haluat tuoda esiin kun puhutaan työstä epidemian aikana?		difficulties caused by Corona epidemic?<br>What do you think about social security schemes compensating for the loss of income among entrepreneurs including Uber drivers?<br>Have you applied for from your company's Corona (Uber/Foodora/Wolt) support schemes e.g. sick leave policy, masks or disinfectants?<br>How do see different support schemes for the difficulties caused by Corona epidemic?<br>Do you know Uber drivers (Foodora / Wolt couriers) who have been infected or have been quarantined?<br>How have they told others and Uber about their illness or quarantine?<br>How common is it that people do not to tell about these? Do you know that happened?<br><br>What do you want to bring up when talking about work during an epidemic?
**Hyvinvointi ja terveys**<br><br>Mitkä ovat suurimpia huoliasi työhösi liittyen? (Jatkokysymykset asiakkaan vastaukseen liittyen; Onnettomuudet, oma terveys, uhkaavat/vaaralliset	**Благополучие и здоровье**<br><br>Что вас больше всего беспокоит в вашей работе?<br>• Несчастные случаи,<br>• личное здоровье,<br>• опасные клиенты,<br>• споры с властями	**Wellbeing and health**<br>What are your biggest concerns about your job?<br><br>(Follow up to informant's answer: Accidents, personal health, dangerous customers, disputes with authorities)<br><br>If your illness prevents you from working, what do

asiakkaat, kiistat viranomaisten kanssa)<br><br>Jos sairaus estää työnteon, miten toimit? (työterveyspalvelun organisointi)<br><br>Miten sinun sairausaikasi korvataan<br><br>Kuinka taksikuskin palkka riittää omaan toimeentuloon tai perheen elättämiseen? (Venäjäksi suora kysymys mikä on viikon/kuukauden palkka)<br><br>• kuinka paljon tunteja keskimäärin viikossa.<br>• Oletko työssä myös jossain muualla?<br><br>Miten sinulla riittää aikaa ja voimia perheelle ja harrastuksille? (työn ja perheen yhteensovittaminen)<br><br>Mihin vuorokaudenaikaan teet työtä yleensä? Miksi juuri silloin?<br><br>Miten näet tulevaisuutesi? Entä Taksialan tulevaisuuden?<br><br>Mitä teet, jos sinua väsyttää ja pitäisi vielä jatkaa työvuoroa?<br><br>Mitä teet, kun joudut uhkaavaan tilanteeseen (fyysinen väkivalta)?<br><br>Voitko kertoa esimerkin merkittävästä uhka- tai ristiriitatilanteesta asiakkaan kanssa (Venäjällä	Что вы делаете, если оказались в опасной ситуации на работе (если есть угроза насилия)?<br><br>Расскажите о последнем случае, когда вы оказались в стрессовой ситуации на работе или у вас был конфликт с клиентом? Что вы предпринимали и как ситуация была решена?<br><br>Если вы заболели и болезнь мешает вам работать, что вы делаете? (организация медицинского обслуживания)<br><br>Ваша зарплата в такси достаточна, чтобы содержать себя или свою семью? Сколько вы зарабатываете в неделю/месяц?<br><br>• про рабочее время, спросить, сколько часов в неделю в среднем работает в такси<br>• Есть ли другие места работы<br><br>У вас остается время и силы для вашей семьи и хобби?<br><br>Каким вы видите свое будущее? Будущее такси?	you do? (organization of occupational health service)<br><br>Do you get compensation for that time/sickness pay?<br><br>Is the taxi driver's salary sufficient to support himself or his family? (In russian direct querstion about weekly/monthly salary)<br><br>(If informant refers to working hours, you may ask how many hours per day/week on average he or she works)<br><br>After your work how much time do you have and energy for your family and hobbies?<br><br>What time of the day do you usually work? Why this time?<br><br>How do you see your future? What about the future of taxi?<br><br>What do you do if you feel tired but you have to stay on your shift?<br><br>What do you do in threatening situations / facing the threat of physical violence? Could you tell me about the last threatening situation?

epäselvyys kuljettajan ja asiakkaan pelisäännöistä)?		
**Maahanmuuttajat**<br><br>minkälaiset välit sinulla on muiden kuljettajien kanssa?<br><br>miten ”etninen tausta” vaikuttaa taksikuskina toimimiseen? (sopivat lisäkysymykset jos tarpeen)<br><br>Mitä huolia tai haasteita sinulla on poliisin tai viranomaisten suhteen?	**Для этнических меньшинств и мигрантов**<br><br>• какие у вас отношения с другими водителями Яндекс?<br><br>- Как ваше «этническое происхождение» сказывается на работе?<br><br>- Можете ли вы привести пример хорошего опыта работы с клиентом?<br><br>- Какие проблемы или сложности у вас есть в отношениях с властями, полицией, гос органами?<br><br>- Что вы думаете о публичных обсуждениях о местных и не местных водителях Яндекса?	**Immigrants**<br><br>- what kind of relationship do you have with other drivers?<br><br>- How does the "ethnic background" affect taxi driver behavior? (Ad hoc)<br><br>- What concerns or challenges do you have with the police or authorities?<br><br>- What do you think about the public debate about immigrant drivers?
**Nuoret**<br><br>miten näet taksikuljettajan työn osana nykyistä elämäntilannettasi?<br><br>missä ammatissa näet itsesi tulevaisuudessa?<br><br>itä ajattelet koulutuksestasi?<br><br>- Aiotko vielä opiskelemaan? Jos kyllä, niin mitä aiot opiskella?<br><br>Miksi juuri sitä alaa?	**Молодежь**<br><br>Лакое место работа в такси занимает в вашей сегодняшней жизни? (Как вы видите работу водителя такси как часть вашей текущей жизненной ситуации?)<br><br>Каким вы представляете свое будущее? Чем вы планируете заниматься в будущем?<br><br>В какой профессии вы видите себя в будущем?<br><br>Что вы думаете о своем образовании?	**Young people**<br>- How do you see the work of a taxi driver as part of your current life?<br><br>- how do you see your future?<br>- In what profession do you see yourself in the future?<br>- what do you think about your education?<br>- Are you still going to study? What and why?

	Вы все еще собираетесь учиться? чему и почему?<br><br>Я исследователь и не контролирую вашу работу, вы могли бы показать вашу путевой лист? Вы перед каждой сменой проходите медосмотр и технический контроль?	
